# Patient and Public Involvement and Engagement in a doctoral research project exploring self‐harm in older adults

**DOI:** 10.1111/hex.12917

**Published:** 2019-05-26

**Authors:** M. Isabela Troya, Carolyn A. Chew‐Graham, Opeyemi Babatunde, Bernadette Bartlam, Adele Higginbottom, Lisa Dikomitis

**Affiliations:** ^1^ Research Institute for Primary Care and Health Sciences Keele University Keele UK; ^2^ Midlands Partnership Foundation Trust Stafford UK; ^3^ West Midlands Collaboration for Leadership in Applied Health Research and Care Coventry UK; ^4^ Family Medicine and Primary Care Lee Kong Chian School of Medicine, Nanyang Technical University Singapore Singapore; ^5^ School of Medicine Keele University Keele UK

**Keywords:** aged, patient participation, qualitative, self‐harm, self‐injurious behavior, systematic review

## Abstract

**Background:**

The contribution of involving patients and public in health research is widely reported, particularly within mental health research. Less is written about such contributions to doctoral research. The research focus of this doctoral research, self‐harm in older adults, was put forward by a Patient Public Involvement Engagement (PPIE) group, who contributed to its development.

**Aims:**

Critically reflect on the process, potential impact and identify challenges and opportunities in involving robust PPIE in a doctoral study.

**Methods:**

Three PPIE members contributed to a systematic review (SR) and a qualitative study through a series of four workshops to meet the aims of the study. PPIE contributed to developing the SR review questions, protocol, data analysis and dissemination of findings. For the qualitative study, they helped develop research questions, protocol, public‐facing documentation, recruitment strategies and data analysis. Involvement followed the GRIPP2‐SF reporting checklist.

**Results:**

PPIE enhanced methodological rigour, data analysis, interpretation and dissemination of findings. Challenges included lack of ethical guidance, time‐related pressures and ensuring support for PPIE members. These were successfully managed through ongoing dialogue and regular communication.

**Conclusions:**

PPIE can enhance the quality and depth of doctoral research, as lived experiences shared by PPIE members add to research's components. Exposing early‐career researchers to PPIE can build research cultures sensitive to PPIE's potential contribution and develop the expertise needed to avoid tokenistic involvement. Capturing lay perspectives is essential in mental health research to ensure research findings are accessible and that findings inform clinical practice. However, clear guidance on the ethical dimensions to PPIE is needed.

## 1 BACKGROUND

The involvement of patients and the wider public in health research has been reported increasingly over the last decades. The rationale for including a patient and public perspective across a range of research methodologies, including systematic reviews (SR) and qualitative studies, has been advocated by leading health authorities such as the United Kingdom's (UK) National Institute for Health Research advisory group INVOLVE.^1^ Within SRs, for example, including patient perspectives has shown to improve the quality of studies and relevance of findings to patients.^1^, ^2^, ^3^ Despite these benefits, the reporting of Patient Public Involvement and Engagement (PPIE) in SRs is still scarce.^4^ In contrast, evidence of the benefits of PPIE in qualitative health research has been increasingly documented.^5^, ^6^, ^7^, ^8^

Within the field of mental health, research conducted in collaboration with the public has gained popularity due to its potential of enhancing quality and appropriateness of research, improving engagement of interventions, alongside the gained service‐user perspective contributing to the acceptability and applicability of research.^5^, ^9^, ^10^, ^11^, ^12^ Several challenges have been reported when conducting research with vulnerable populations, for instance those living with mental health problems, such as difficulty reaching participants, lack of engagement and difficulties in capturing insider perspectives. Such challenges could be mitigated by including the patient perspective from early in the research process.^9^, ^10^

In the UK, the country where this study was conducted, PPIE is now a prerequisite for many funding bodies, but is not a requirement for doctoral studies, which may result in a lack of PPIE in the work of early‐career researchers. Reported challenges for PPIE involvement include lack of researchers’ engagement and involvement, which could be mitigated by incentivising early‐career researchers to include PPIE in their research.^5^ Furthermore, many doctoral studies are unfunded, resulting in an added difficulty to PPIE involvement as there may be no funding for PPIE activities. The aim of this paper was to critically reflect on the process, potential impact and identify challenges/opportunities in involving robust PPIE in a doctoral research, including a SR and qualitative study.

The concept of this research arose from an earlier project on self‐harm in primary care,^13^ which was undertaken in collaboration with a PPIE group. As an outcome of that study, PPIE members noted the importance of investigating self‐harm in older adults. This is a population which is often overlooked, yet recent studies suggest self‐harm in older adults results in increased mortality compared to younger groups.^14^, ^15^ The group contributed to developing the idea as a doctoral research proposal and funding application, resulting in the doctoral research project presented here. The research consisted of two components: a SR and a qualitative study. Brief summaries of the research questions, project design, methods and results of the two studies are presented in Boxes 1 and 2 .

BOX 1 **Summary of Methods and Results of the systematic review of self‐harm in older adults**

**Research question**

What are the main characteristics (rates and risk factors) of older adults who self‐harm, including clinical characteristics and lived experiences of self‐harm?

**Methods of the systematic review of self‐harm in older adults**

A comprehensive search strategy was used to search five e‐databases.

Key *inclusion criteria* were as follows: (a) population: studies examining older adult populations (aged 60 years or older) with presence of at least one self‐harm episode as defined by NICE guidelines. (b) Exposure: self‐harm determined by clinical presentation, self‐report, or reports from family, carers, or health practitioners regardless of suicidal intent. (c) Outcomes: studies reporting at least one clinical characteristic (eg self‐harm rates, methods, risk factors and repetitions) and/or lived experiences with self‐harm. (d) Study designs: observational studies with or without comparison groups from both clinical and community populations.

*Exclusion criteria* were narrative reviews, letters, editorials, commentaries and non‐English language studies for which interpretation could not be obtained.

The *methodological quality* of studies was independently appraised by pairs of reviewers.

Results from included manuscripts were summarised using thematic analysis and synthesis.

**Results of the systematic review of self‐harm in older adults**

**Figure 1:**
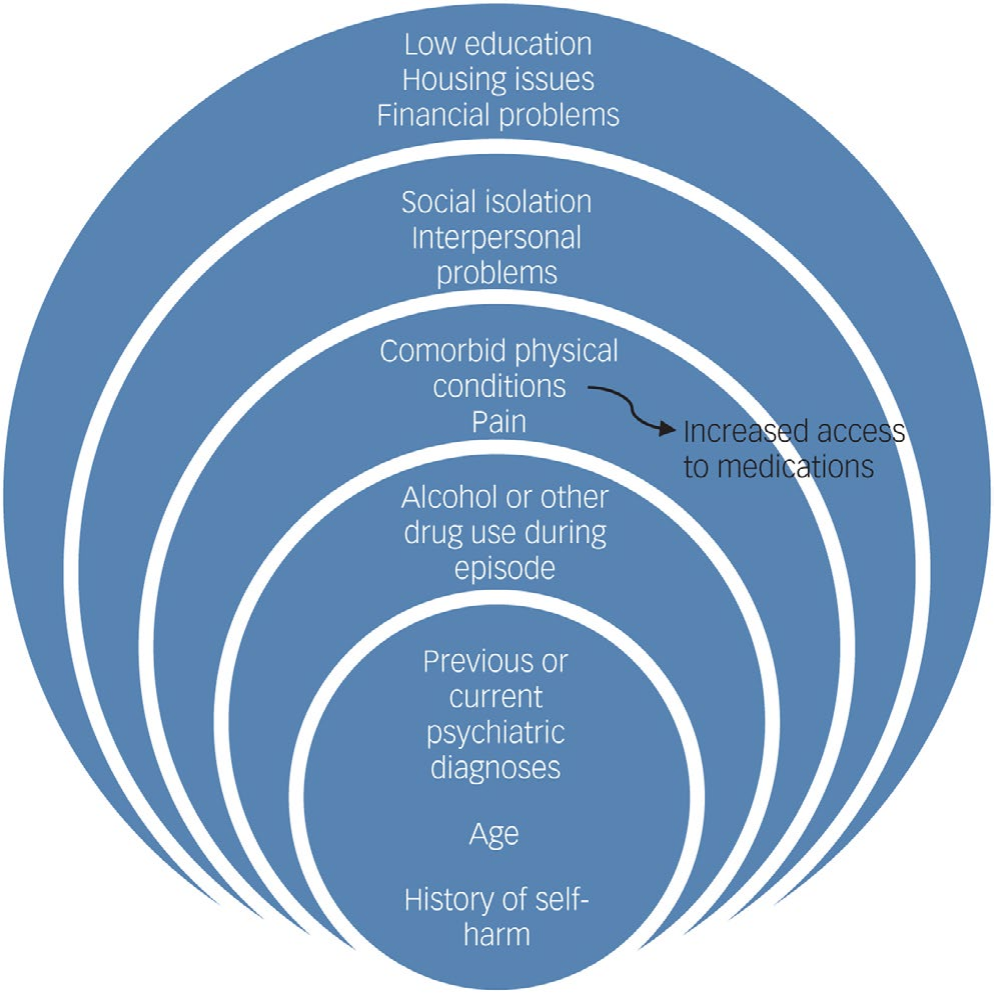
Influencing factors for self‐harm in older adults (reproduced with permission from Cambridge University Press, Troya et al^15^). + Diagram presented in layers according to internal and external factors. Different size layers do not refer to higher or lower association to self‐harm but rather represent internal and external factors

Forty studies met inclusion criteria. Previous history of self‐harm, previous and current psychiatric treatment and socio‐demographic factors (single, living alone and younger older adults aged 60‐74 years old) were found to be significant risk factors for self‐harm repetition. Others, such as alcohol/drug use, psychiatric history and a diagnosis of musculoskeletal conditions such as arthritis were also associated with self‐harm repetition but the overall quality of evidence for these factors ranged from low to very low. A thematic analysis of the influencing factors for self‐harm in older adults is summarised in Figure 1. Influencing factors range from internal (eg age, gender) to external factors (eg financial worries, low education), showing the complex relationship between these factors throughout the presented layers. Loss of control, increased loneliness and perceived burdensome ageing were reported self‐harm motivations.

BOX 2 **Summary of Methods and Results of the qualitative study on self‐harm in older adults**

**Research questions**

What are the perceived motivations for self‐harm in older adults?

What are the barriers and facilitators of access to care and support?

What are the potential roles, if any, of family, friends, third sector and primary care in supporting older adults who self‐harm?

**Methods of the qualitative study on self‐harm in older adults**

Semi‐structured interviews were held with older adults (≥60) who engaged in self‐harm and third‐sector support workers in England. Older adults were invited to consent to a follow‐up interview to be conducted one to three months after the first interview in order to allow further discussion, reflection and analysis of the first interview. *Inclusion criteria* for older adults were current or previous self‐harm history (within the age of 60); third‐sector support workers having previous experience working with older people who self‐harm. All participants needed to be fluent in English to be eligible. Participants were recruited from third‐sector groups, online advertisement and advertisement in North Staffordshire. Interviews were recorded, transcribed verbatim and data analysed using thematic analysis and constant comparison methods.

Ethical approval was obtained from Keele University's Ethics Review Panel (REF: ERP1333).

**Results of the qualitative study on self‐harm in older adults**

Between September 2017 to September 2018, 24 interviews were conducted involving 16 participants. All older adults had a diagnosis of mental illness in addition to a physical illness. Different identified stressors experienced throughout the life‐course left older adults in a vulnerable position where self‐harm was used to manage distress. Stressors included adverse events, loss, interpersonal and health problems. Shame and stigma were experienced by older adults.

**Interpretation of findings of the qualitative study on self‐harm in older adults**

Self‐harm was experienced within a suicidal spectrum of no‐suicidal intent to attempted suicide, providing evidence of self‐harm being non‐static and evolving throughout the life‐course. Findings suggest that the relationship between self‐harm and future repetition is more complex given that in some older adults, engaging in self‐harm allowed them to avoid suicide. Self‐harm is well concealed in older adults given high levels of stigma experienced within this population, which may lead older adults not reporting self‐harm or seeking support. Older adults with comorbid health conditions should be adequately assessed for risk of suicidal behaviour.

## 2 METHODS

We undertook a critical reflection on the research process, challenges encountered in involving PPIE and its impact on the doctoral study. To do this, we reflected on the research as a whole, the aim of PPIE, who group members were, the support offered to them and details of how involvement was structured. The reflection is based on the minutes of research meetings and PPIE workshops, which documented discussions on changes to the project resulting from PPIE, together with a review of the PowerPoint slides presented to the group. To document the involvement of PPIE in this doctoral research project, the reporting checklist GRIPP2‐SF (Guidance for Reporting Involvement of Patients and the Public‐Short Format) was followed (Appendix 1).^16^

PPIE members of the previous study^13^ who had suggested the topic of the doctoral research and who met inclusion criteria were invited to join the new PPIE group. Inclusion criteria were having previous lived experience with self‐harm as an older adult (60 years or older) and/or have worked with (eg support worker) or cared for (eg carer) an older adult with self‐harm behaviour. PPIE involvement in the study was supported by the PPIE team at Keele University, which has over a decade of experience involving patients and the public in health research. The structure and operations of the PPIE team within the Research Institute have been reported elsewhere.^17^ Individuals from the previous PPIE group were invited to participate in the present research. Interested individuals and consequently the PPIE group for this research consisted of an older female adult with self‐harm history, a male carer and a female support worker with previous experience of self‐harm. All members were aged 60 and over. The decision not to look for any further PPIE members was made by the research team considering the importance of maintaining and sustaining a PPIE group throughout the 3‐year duration of the study, and the group already offering the range of expertise needed, particularly in light of members’ previous PPIE experience. Regular communication was put in place, including quarterly updates on the PhD project and a feedback postcard after each workshop summarising PPIE contributions and any changes undertaken as a result.

### 2.1 PPIE training and workshops for PPIE members

A total of four workshops were held at different stages of the research, in order to work simultaneously on both the SR and qualitative study. Duration of workshops varied from two to three hours, with half of the time in each allocated to the SR and the other half to the qualitative study. All workshops were held at the University, a location familiar to group members because of their participation as PPIE members in the previous study.^13^ Travel reimbursements and vouchers for compensation of time spent were provided after each workshop following current guidelines.^18^ Before each workshop and at regular intervals throughout, PPIE members were asked verbally if they felt comfortable and able to continue participating. On no occasion did any PPIE member decline to continue, with all three members attending the four workshops. Before each workshop, the research team discussed areas to be covered.

The research team took key decisions regarding the role and level of involvement of the PPIE group, including the decision not to include members as co‐researchers/co‐interviewers because of concerns not to cause undue emotional upset. Further discussions took place with the group to clarify roles and refine levels of participation in order to avoid overburdening. PPIE members did not offer suggestions with regard to the structure of their involvement. Both of these concerns were rooted in ethical issues around the well‐being and safety of PPIE members. To record the impact of the involvement and contribution of PPIE members in the study, the team documented the changes made and overall contribution after each of the workshops and fed this back to PPIE members. Conversations held amongst the research team allowed for the analysis and consideration of PPIE contribution to the study. Furthermore, there were opportunities for the group (PPIE group, researchers and PPIE coordinator) to reflect on the research project and process.

Throughout the different stages of involvement, brief training (on the topic and methods of enquiry) and support were given to PPIE members. Support (logistical, training and well‐being) was provided by the PPIE coordinator (AH), in addition to the attending research members (IT and/or CCG). Logistical support included ensuring meeting venues were accessible to members, as well as coordinating meetings at a date and time convenient for PPIE members. Training support entailed lay friendly and accessible materials explaining the details of the research project. Support for well‐being included enabling members to feel they could speak freely within workshops and ask questions of any kind and careful observation of members’ emotional and physical needs (ie presenting data sensitively, ensuring adequate breaks for refreshments). Mechanisms were put in place so that if any distress associated with being a PPIE member was noted, this could be addressed appropriately. Two research members (IT and CCG) have clinical backgrounds in mental health, and it was anticipated that one of them would speak privately with the individual, check the nature of distress and identify with them appropriate ways forward. Broader implications would be discussed with the wider team to identify possible implications for PPIE generally and additional support strategies for the group if appropriate. However, no situations of distress were encountered.

Regarding researchers’ well‐being, after each workshop and throughout the study duration, research members had the opportunity to discuss sensitive and potentially upsetting matters with the rest of the research team. Although no distress was encountered, discussions held with the rest of the research team were helpful to avoid such potential distress, in addition to the research members involved having clinical backgrounds.

We now present full details of the workshops, first in terms of contribution to the SR and then to the qualitative study.

#### 2.1.1 Systematic review

At the first workshop, members from the PPIE group and research team were introduced and an outline of the doctoral research project was presented. The presentation was followed by a discussion of different definitions used in the research literature. PPIE members also deliberated on set eligibility criteria for the SR. However, SRs are a very specific and complex approach to synthesising evidence, and it became apparent in the workshop that more information regarding the process of conducting SRs needed to be provided to PPIE members in order for them to make a meaningful contribution.

Consequently, in the second workshop, the concept and process for undertaking SRs were presented and defined in detail. The different stages of SRs were explained, alongside the overall purpose and contribution of this approach. Time was given for discussion and questions. The results of the initial search strategy and index papers (*n = *4) to be included in the review were also presented along with instruments for data collection (data extraction sheet and quality assessment toolkits) to ensure comprehensive capture of data items relevant to the SR. An a priori protocol was subsequently established and registered on PROSPERO, an international prospective register of systematic reviews: CRD42017057505.

During the third workshop, results from the final search strategy were presented and discussed to seek PPIE members’ views and interpretations. Members also contributed to the thematic synthesis from the included qualitative studies.

In the fourth and final review workshop, advice from the group was sought on dissemination of the SR findings and ways to maximise impact.

#### 2.1.2 Qualitative study

PPIE members’ opinions were considered in the planning of the study design. In the first workshop, the proposed research questions (informed by the previous PPIE group) were presented to members for feedback. An outline of the proposed data collection methods, as well as public‐facing documentation, was also presented. Given the scale of the anticipated contribution of the PPIE group to all aspects of the work, this first meeting was held prior to submitting the study proposal to ethical review.

During the second meeting, members were updated regarding the progress of the study and issues around participant recruitment were considered in detail, including appropriate ways of reaching potential participants.

The third meeting explored difficulties in recruitment and possible alternative strategies for identifying potential participants. Also discussed were timelines for fieldwork, data generation and analysis. Methods for analysing qualitative data were briefly presented, concentrating on the analytical approach to be used when conducting the research.

The fourth workshop was used to discuss ongoing challenges encountered whilst recruiting participants. Data from interview transcripts were also presented and members invited to contribute to their analysis.

## 3 RESULTS

The results of PPIE in this doctoral research project are presented and discussed below, first in terms of the SR, followed by the qualitative study. Building on previous research,^3^, ^19^, ^20^ Table 1 highlights the challenges encountered throughout the research process when involving PPIE, as well as suggestions for researchers looking to adopt PPIE in their research. In Table 2, examples for both the SR and qualitative study are provided regarding the changes made after PPIE involvement in the research.

**Table 1:** Challenges and suggestions when involving PPIE in a doctoral research project

Challenges^3^, ^19^, ^20^	Implications of unresolved challenges	Strategies used	Suggestions for encountered challenges	Considerations and suggestions when involving at risk populations
Time‐related pressures	Superficial involvement resulting in lack of meaningful impact	Liaised with the Institute's established PPIE network 8 months prior to the first meeting, contact with PPIE coordinator was made in order to have sufficient time for involving patients and logistic of PPIE involvement	Consideration of PPIE involvement from early stages of planning research (ideally when preparing funding application) Allocate time for possible delays; realistic deadlines Liaise with PPIE network and have lead PPIE coordinator	Defining clear roles and responsibilities Prioritising PPIE's well‐being Offering adequate support and training Acknowledging contributions Promoting PPIE throughout the research cycle Offering accessible and inclusive approaches tailored for the specific PPIE members
Resources (time and funding)	Potential burden caused to PPIE members Superficial involvement due to insufficient time and financial consideration	Secured separate funding for PPIE activities, including engagement Liaised with PPIE coordinator for reimbursement of members’ involvement Researcher allocated time for PPIE activities	Early consideration of PPIE involvement in order to plan and allocate enough time and funding Liaise with PPIE network Use available resources for guidance and templates of PPIE^1^ Offer reimbursement to PPIE members Allocate enough time for PPIE involvement	
Avoiding tokenistic involvement	Lack of meaningful impact and PPIE ‘tick box’ approach used for funding applications resulting in superficial involvement No careful consideration of involvement of PPIE's time and potential contribution	PPIE workshops planned in multiple times of the research project After each workshop, detailed account of impact of PPIE documented and shared with research team and PPIE members Sufficient time and space given to PPIE group to allow for in‐depth discussion	Keep clear and accessible records of PPIE involvement throughout the different stages of the research project Clarify involvement and level of involvement by each of the members Value the involvement, contribution, added perspective given by PPIE Liaise with research team and PPIE coordinator to ensure meaningful involvement	
Continuity	Discontinued involvement resulting in superficial PPIE Disregard of PPIE's potential contribution and time	PPIE workshops and objectives planned in advance and discussed with PPIE group and coordinator Feedback PPIE members regarding stage of research Encourage participation through thoughtful consideration of PPIE needs and capacity of involvement	Provide clear expectations, define roles and responsibilities, involvement timelines Ensure PPIE's needs are considered and involvement is not resulting in burdening members Ensure feedback is provided regarding PPIE's impact and contribution to the study, as well as stage of study Acknowledge the different needs PPIE members may have which can limit their ongoing involvement	
Clear and open communication	Unrealistic expectations and management of roles which can lead to disengagement of PPIE	Liaised with PPIE coordinator in order to maintain regular communication and ensure lay friendly language used in workshops	Liaise with PPIE network to ensure clear, open, accessible and bilateral communication Use available resources for guidance and templates of lay friendly language (www.invo.org.uk/) Provide different communication avenues (post, email, phone)	
Working sensitively	Burdening PPIE members and potentially causing distress Misrepresentation of diversity of PPIE group	Open conversation regarding participants needs (both physical and emotional needs) Ensure PPIE group involvement can discontinue at any time individual chooses Liaised with PPIE coordinator and research team to ensure power balance in group dynamic was achieved in order to represent the diversity within the PPIE group Flexibility in dates and times PPIE members could attend meetings	Presenting material sensitively and cautiously Offer avenues of support if needed (eg GPs) Ongoing consideration of physical and emotional needs Ensure members involvement can stop at any point they wish Acknowledgement and consideration of different group dynamics and power balance amongst PPIE members Awareness of ethical issues around safety and well‐being	
Recognition of public involvement	Disempowerment and disregard of PPIE's contribution	Reimbursement of expenses Openly asked members how they would like to be acknowledged in presentations, publications, etc	Liaise with PPIE network regarding expense guidance Have a clear conversation regarding level of involvement and capacity of PPIE members as well as confidentiality and anonymity possible needs when acknowledging involvement Be prepared for inclusive involvement of members in engagement activities	

**Table 2:** Examples of PPIE involvement and influence on PhD research project

Research element consulted/discussed	Study	Workshop	Before workshop	Impact after PPIE involvement
Definitions to be used	SR and Qualitative	Workshop 1	Possible definitions: Attempted suicide, Non‐suicidal Self‐Injurious Behaviour (NSSI), self‐harm behaviour Older adult: 40 y and older	Collectively the decision to use NICE guidelines (CG13) definition for self‐harm was made, highlighting the possibility of self‐harm with and without suicidal intent World Health Organization's definition of older adult adopted: 60 years and older
Research questions	Qualitative	Workshop 1	Exploring the role of the formal sector in supporting older adults with self‐harm behaviour Understanding motivations to self‐harm in older adults	Role of the informal sector in supporting older people who self‐harm The identification of barriers to self‐harm in addition to motivations
Methods	Qualitative	Workshop 1	Interview older adults and general practitioners One interview with participant groups	Interview older adults and third‐sector support workers Addition of a follow‐up interview with older adults
Public‐facing documentation	Qualitative	Workshop 1	Information sheet, interview guide draft provided to PPIE group	The word dissemination was removed from the information sheet as it was not lay friendly The group commented on the order of items for discussion in the interview guide, suggesting starting off by asking demographic and clinical questions prior addressing motivations of self‐harm. As well, members suggestion of including triggers to self‐harm was incorporated
Search strategy	SR	Workshop 2	Search strategy including different terminologies used for self‐harm and older adult	Self‐destructive and pensioners added to search strategy
Data extraction	SR	Workshop 2	Elements of data extraction sheet discussed	Subgroup analysis in the different age ranges of older adults Subgroup analysis amongst non‐suicidal self‐harm and attempted suicide
Recruitment avenues	Qualitative	Workshop 2	Proposed avenues for recruitment: self‐harm support groups, advertising in local community, GP practices, social media	Agreed avenues of recruitment: self‐harm support groups, age support groups, female support groups, advertisement in local community, social media
Identification of limitations of SR	SR	Workshop 3	To be discussed in workshop	Majority of evidence from hospital‐based settings, ‘younger’ older adults Identification of gaps in the literature: alternative methods of self‐harm (eg overeating)
Interpretation of findings from SR	SR	Workshop 3	Presentation of quantitative and qualitative data to the PPIE group	Socio‐demographic characteristics: likelihood of more ‘younger’ older adults captured in review due to ‘older’ older adults dying as a result of self‐harm due to frailer health Suicidal intent: although majority studies reporting attempted suicide, in reality suicidal intent is unclear even to patient. Accidental deaths caused due to self‐harm
Difficulties in recruitment	Qualitative	Workshop 3	Discussion of difficulties in recruitment due to low‐participation rate	Suggestion of IT attending self‐harm support groups regularly so potential participants can feel more comfortable in being part of study. This reiterated discussions amongst the research team and resulted in increased participation
Dissemination of findings	SR	Workshop 4	Information leaflet draft presented to PPIE group	Alongside NICE definition of self‐harm, another lay friendly self‐harm definition Sources of support identified: helplines for older adults and GPs Identification of avenues to disseminate findings: GP practices, libraries, retirement homes and third‐sector services
Interpretation of qualitative findings	Qualitative	Workshop 4	Quotes presented to participants in order to identify initial themes	Themes suggested by PPIE group: difficulty asking for help due to shame in older adults, self‐harm used as a coping method, difficulty stopping self‐harm, self‐harm due to different life‐course stressors. These themes were conserved and only slight medications of wording resulted after consulting with the rest of the research team
Ethical considerations in recruitment	Qualitative	Workshop 4	Examples of challenging situations in recruitment were presented to the group to seek feedback	The PPIE group provided tips for encountering challenging situations in recruitment: (a) ensuring to state clearly from the start the age range and self‐harm definition used as eligibility criteria; (b) discuss with support workers any difficulties encountered with participants wanting to engage in the study that were not eligible

### 3.1 Systematic review

#### 3.1.1 Workshop 1: Refining the scope of the review

PPIE members influenced the scope of the SR by refining the overall aim and definition of key terms (self‐harm and older adults). The main difference amongst existing definitions of self‐harm consists of the presence or absence of suicidal intent when engaging in self‐harm (non‐suicidal self‐injury vs attempted suicide).^21^ After considering the strengths and limitations of the existing definitions, members reached consensus and selected the National Institute for Health and Care Excellence (NICE) Guidelines^22^ definition for self‐harm: ‘any act of self‐injury or self‐poison irrespective of apparent purpose of the act’. Reflecting discussions within the research team, the group were also asked to consider how best to define older adults (eg age criteria). It was agreed to use the World Health's Organization definition of any person aged 60 and over.^23^

#### 3.1.2 Workshop 2: Introducing systematic reviews, search strategies and outcomes

In the second meeting, the initial draft search strategy was refined by the group to include additional terms (see Table 2). PPIE members also helped refine the inclusion and exclusion criteria, to exclude or perform subgroup analyses of studies reporting self‐harm with exclusive suicidal intent. As mentioned above, in workshop 1, the group had agreed on an overall focus of self‐harm (including both suicidal and non‐suicidal intent). However, when presented with the distinction in the literature between those studies using the term ‘non‐suicidal self‐injury’ and those using the term ‘attempted suicide’, the group decided that clarification in the analysis and interpretation of findings was needed around the different self‐harm definitions used by studies.

Lastly, regarding data extraction and analysis, PPIE involvement led to the addition of other self‐harm outcomes related to alcohol and drug use of participants, as well as subgroup analyses according to age groups of older adults (ie ‘younger older’ adults: 60‐74; ‘older older’ adults 75 and over).

#### 3.1.3 Workshop 3: Analysis and interpretation of SR findings

Members identified limitations to the SR findings, particularly around the representation of younger older adult age groups and also methods of self‐harm reported amongst the studies. Findings from the SR showed that available evidence was mostly from hospital‐based settings in which those with more severe outcomes were cared for. This led PPIE members to speculate on the likelihood of self‐harm amongst older adults being under‐reported. The group suggested that self‐harm presentations using other less fatal methods such as self‐injury were likely to be under‐represented in clinical presentations to health services and therefore not captured by the SR.

A conceptual framework (Figure 1) was developed from the different identified themes emerging from the qualitative data of the SR. From the initially presented themes (*n* = 16), the group clustered these into three overarching themes: loss of control contributing to the suicide attempt, increased loneliness and isolation and ageing perceived as ‘burdensome’ and affecting daily living.

The group also identified gaps in the literature from the SR which they considered as important for patients and public and which require further research. These included alternative methods of self‐harm (eg overeating, alcohol abuse), studies focusing on self‐harm reported in community settings and the role of carers in supporting older people who self‐harm. The group's interpretations and feedback were taken into account by the research team in conceptualising findings from the review.

#### 3.1.4 Workshop 4: Dissemination of findings and pathways for engagement

The final workshop in which the SR was discussed focused on identifying strategies to disseminate findings from the SR with members of the wider public. The group co‐designed an information leaflet (Appendix 2) based on the review's results. As part of this, in addition to the already identified NICE guidelines definition of self‐harm, PPIE members added another commonly used definition which they considered to be more understandable to a lay audience (‘a coping mechanism that is harmful to a person's wellbeing’). PPIE members also suggested including additional sources of support based on their own experiences (helplines for older people and general practitioners).

In addition to contributing to the content and format of the leaflet, the group also suggested venues where the information leaflet might be made available in order to be more accessible to at risk individuals and those supporting them (eg pharmacies, GP practices, libraries, retirement accommodation and third‐sector services). Lastly, a discussion took place on other dissemination activities such as developing abstracts, presentations and publications, to reach consensus on the best approach to ensure recognition of the group's contributions, whilst protecting privacy.

### 3.2 Qualitative study

#### 3.2.1 Workshop 1: Defining aims and methods of qualitative study

In addition to agreement on definitions, members identified different factors which may be of importance and relevance when researching the experiences of older adults who self‐harm. Members critiqued and added to the overall research questions of the study and ensured these were service‐user focused as reflected in Table 2.

The group was asked their views on whether to include other participant groups (eg third‐sector workers). Members supported the idea of including third‐sector workers as they felt that interviewing this group was important, given their frequent contact with older adults who self‐harm. Furthermore, the group confirmed appropriateness of additional follow‐up interviews with older adults, as they considered this would aid rapport building and trust between researcher and study participants.

Lastly, the group contributed to refining participant‐facing documents, including study posters, topic guides, information leaflets and consent forms. Members confirmed the proposed content areas for the topic guide (Appendix 3). Some of the changes made in these documents are summarised in Table 2.

#### 3.2.2 Workshop 2: Refining recruitment strategies

Members identified the likely difficulties in using one of the proposed recruitment avenues (community groups, not specific to provide support for self‐harm) and considered that some older adults may not self‐identify and/or be unprepared to discuss their self‐harm. The group suggested alternative methods of recruitment to help reach the targeted population: local third‐sector groups for older people, as well as female‐specific support groups and mental health charities. These suggestions confirmed discussions taking place within the research team. Members’ alternative suggestions of recruitment strategies were adhered to following considerations by the research team.

#### 3.2.3 Workshop 3: Preparation for data analysis

A brief introduction to data analysis was provided as preparation for meeting 4 when transcripts would be analysed. Alternative recruitment strategies were discussed with the group given the initial low‐participation rate. PPIE members suggested that IT should attend the local self‐harm support group regularly, as they felt this would make potential participants feel more comfortable when approached to participate in the study. Once again, this reflected and supported discussions within the research team. Following these suggestions seems to have resulted in increased participation.

#### 3.2.4 Workshop 4: Analysis and interpretation of findings of the qualitative study

Collaboratively, the group identified initial themes emerging from the data, as well as initial grouping of codes and categories. All views were considered and incorporated into the analysis. The input to the analysis contributed lay perspective to interanalyst consensus/triangulation of the data,^24^ increasing the potential relevance for older adults who self‐harm. In several instances, the group provided an additional interpretation and understanding of initially proposed themes and explanations of the data as can be seen in Table 2 (eg different self‐harm methods used by participants according to the varying stimulus to self‐harm). Lastly, discussions held about difficulties encountered in recruitment and interviewing were helpful as feedback was received from the group with how best to handle challenging situations.

## 4 DISCUSSION

This article presents an account of how a robust collaboration between a PPIE group and research team contributed to the development of a doctoral research project, including a SR and qualitative study, which found its inception in the recommendations of a previous PPIE group's input in a self‐harm study. To our knowledge, this is the first report to (a) critically review PPIE involvement in doctoral research amongst potentially vulnerable populations, in this case older adults with self‐harm behaviour; (b) provide useful insights into the importance of early‐career researchers operationalising PPIE; (c) make useful suggestions about overcoming PPIE barriers and optimising its benefits; and (d) state the importance of having greater engagement with ethical implications.

Through a series of four workshops, PPIE contributed to refining the scope of the SR, revising definitions, search terms and outcomes to be used, as well as the analysis and interpretation of findings through the development of a conceptual framework of influencing factors of self‐harm in older adults and the elaboration of a lay friendly information leaflet. In the qualitative study, the group's input resulted in modification of recruitment strategies and methods for data collection, resulting in a richer data set, ensuring a comprehensive capture of populations of interest. Involvement also strengthened the methodological rigour of results, by adding validity through triangulation of the analysis and interpretation of findings.

### 4.1 Challenges and opportunities of involving PPIE in a doctoral research project

Involvement and engagement with PPIE in the research process contributed to improving relevance, legitimacy and validity of findings. Collaboration and ongoing consultation with PPIE in the research process contributed to the added perspective and understanding of study findings, as well as ensuring a broader capture and prioritisation of the public's needs. This study adds to the growing evidence of PPIE's impact and contribution to improving the quality of research projects.^3^, ^4^, ^5^, ^8^

When conducted with adequate support and guidance, PPIE can offer researchers, patients and the public continuity in the research process. Such was the case when conducting this doctoral research project, given the repeated engagement from PPIE members throughout the study. Continuous PPIE involvement was achieved through careful consideration of the PPIE group's capacity, level of involvement, respect of well‐being and adequate training and support.

Drawing upon previously identified frameworks identifying challenges of involving PPIE in research,^3^, ^17^, ^19^, ^20^ Table 1 summarises common challenges when involving PPIE in research, as well as suggestions for researchers. Unresolved challenges could result in superficial involvement, lack of meaningful impact, disregard of the public's potential contribution to the research project, as well as other ethical concerns.

Introducing the theory, ethics and practice of PPIE to early‐career researchers, such as doctoral students, can help create a research culture that values PPIE. Some of the documented challenges for meaningful involvement of PPIE in research include lack of researchers’ engagement and involvement.^5^ We believe that by introducing PPIE to early‐career researchers, such as doctoral students, challenges of meaningful involvement and initial resistance from researchers can be mitigated, as well as contributing to building an early research culture where PPIE is part of researchers' agenda.

Avoiding tokenistic involvement is one of the reported challenges for PPIE as summarised in Table 1. The level of PPIE involvement was carefully considered and discussed amongst the research team, PPIE coordinator and PPIE members. In particular, overburdening PPIE members was a concern when thinking of the level of involvement in this sensitive topic. Through discussions, a balance was reached to ensure meaningful involvement whilst maintaining PPIE members’ well‐being.

More specifically, in doctoral studies, two key challenges for meaningful PPIE involvement are highlighted. Firstly, resources for doctoral research projects. Many doctoral studies are unfunded and/or do not have funding allocated for PPIE, unlike other research projects. This may result in an added difficulty in meaningful involvement of PPIE in doctoral studies. Second, expertise is required for successful PPIE. Doctoral students are often novice researchers, which may require access to expert advice on how to best work sensitively with PPIE members’ needs, including identifying strategies for adequate and tailored support and training, for developing trust and inclusivity.

This doctoral study successfully managed the identified challenges through the strategies described in Table 1. The majority of these strategies consisted on having organizational commitment, funding and infrastructure so liaising with a PPIE coordinator/network is possible, working sensitively around members’ needs, offering adequate support and training, respecting and acknowledging members’ contributions and ensuring continuous communication and involvement throughout the research. Lastly, Table 1 also gives researchers suggestions when working with vulnerable populations in PPIE.

Our study not only reports the involvement of patients and the public throughout the research using the recommended reporting checklist GRIPP2‐SF (Appendix 1), but also identified and made use of other studies reporting best practice for involving PPIE in health research, both for SRs^3^, ^19^ and qualitative studies.^6^, ^20^, ^25^ Reflecting on the process of involvement and impact of PPIE was carried throughout the doctoral research project.

Increasingly, PPIE has been reported amongst populations with health conditions in an effort to gain the added perspective and experiential knowledge of those experiencing the health condition.^5^, ^11^ Self‐harm is not a health condition or disorder, but rather a behaviour that an individual engages in. However, many people engaging in self‐harm report physical and mental health comorbidities, with higher comorbidities amongst older adults.^15^ Considerations of the capacity, level of involvement and unwanted added burden to patients must be taken into account when involving the public in research, but even further careful consideration should be taken with vulnerable populations such as those with self‐harm history. Protection of well‐being for PPIE group members, PPIE coordinator and researchers, must come first when involving patients in research, and thoughtful consideration, support, training and experience must be provided to ensure members’ well‐being.

Although there is a growing body of literature documenting its implementation, PPIE remains an emerging concept in research. Whilst any engagement with patients and the public for the purposes of research requires a deep commitment to the well‐documented principles of biomedical research,^26^ currently, there is no requirement for formal ethical scrutiny of processes for engaging and collaborating in this way. This may leave researchers in a position where they unwittingly fail to consider in full the needs, capacity, level of involvement and required resources prior to approaching or working with PPIE members.^20^ It is fundamental for researchers to thoroughly consider patients’ and the public's needs, capacity, level of involvement and required resources prior to approaching or working collaboratively with PPIE. These issues are further accentuated when researching potentially vulnerable populations, as was the case with this doctoral research project.

### 4.2 Limitations

There were two main limitations. Firstly, the number of PPIE members (*n* = 3) included in this study was small. However, these members belonged to different groups of the population of interest, including support workers, carers and older adults with experience of self‐harm. Furthermore, we believe having a small but closely involved PPIE group aided in achieving equal contribution and representation to the study by all members, as well as continuity. Given the sensitive topic of research, a larger PPIE group could have hindered the involvement and equal representation of all members, as well as risked dropout of PPIE members throughout the duration of the study. Therefore, we believe that the number of PPIE members included in this doctoral research project allowed an in‐depth involvement.

Furthermore, it was also not possible to recruit any PPIE members belonging to minority groups. People from ethnic minorities and lesbian, gay, bisexual and transsexual (LGBT) groups also engage in self‐harm behaviour, which is often hidden.^27^, ^28^ However, PPIE members in this research were all of a white‐British heterosexual background, limiting the voice of these minority groups in the research.

### 4.3 Future research

Further research reporting the involvement of patients and public in health research is needed, particularly transparent documenting of the process and impact of such involvement. Research addressing PPIE involvement with at risk or vulnerable populations is needed in order to report on the context‐specific challenges and opportunities when working with such groups. Future research involving PPIE should report and address possible ethical concerns and document the steps taken to address these. Lastly, further work is needed to document more fully the challenges and opportunities of PPIE in doctoral research.

## CONFLICT OF INTEREST

CCG is editor‐in‐chief of Health Expectations. The authors declare no other conflicts of interest.

## APPENDIX 1 GRIPP2‐short form

**Table:**

Section and topic	Item	Reported on page no.
1. Aims	Report the aim of PPIE in the study	1‐2
2. Methods	Provide a clear description of methods used for PPIE in the study	3‐6
3. Study results	Outcomes: Report the results of PPIE in the study, including both positive and negative outcomes	6‐9
4. Discussion and conclusions	Outcomes: Comment on the extent to which PPIE influenced the study overall. Describe positive and negative effects	9‐12
5. Reflections/critical perspective	Comment critically on the PPIE in the study, reflecting on the things that went well and those that did not, so others can learn from this experience	9‐12

## APPENDIX 3 Topic guide used in qualitative study

### Topic Guide Older Adults

#### Opening

Introduction.

State purpose, motivation and timeline.

Confirm participant has understood and signed informed consent prior beginning interview. If any questions regarding study details or informed consent (confidentiality and anonymity) arise, they will be answered prior the start of the interview and checked again at the end. Throughout prompts such as ‘can you tell me more’ etc will be used.

*Transition:* Let me start by asking you some questions about yourself

*Demographic questions*:

Age

Marital status

Education/occupation

Medical condition (if any)

1. Topic A: Reasons for self‐harm

People self‐harm for different reasons, and I wonder if you would be able to talk about when you first started to do this, and the sorts of things that were going on for you at the time.

- Can you tell me about the first time you self‐harmed? How long has is it been going on? Was there any trigger? (Eg loss of a loved one)
- Could you tell me what role self‐harm has had in your life? (Eg help with coping with difficult situations)
- What reasons would you say there are/were around your self‐harm? Has this changed over time?

*Transition:* I would now like to ask you about your experiences of support with health and social services, and then move on to think about other avenues of possible support, including family and friends.

2. Topic B: Barriers and facilitators in accessing care
a) Formal Health and Social Care
  - Are you accessing any care from the social or health sector at the moment? Could you tell me more about it?
  - Could you tell me about any experience were you accessed care in the health or social services after having self‐harmed?
  - Can you tell me how you felt? How did you feel staff responded to you?
  - Was there any support offered to you after the episode? If yes, could you tell me more about this? How long was the contact? If no, how did that leave you feeling? What sorts of support might you have found useful? In what ways?
  - How would accessing care been easier for you?

*Transition:* I would now like to ask you about your experiences of support with the voluntary sector, including self‐help groups

b) Voluntary Sector
  - Are you accessing any care from the voluntary sector at the moment? Could you tell me more about it?
  - Could you tell me about any experience were you accessed care in the voluntary sector after having self‐harmed?
  - Can you tell me how you felt? How did you feel staff responded to you?
  - Was there any support offered to you after the episode? If yes, could you tell me more about this? How long was the contact? If no, how did that leave you feeling? What sorts of support might you have found useful? In what ways?
  - How would accessing care been easier for you?

*Transition:* I would now like to ask you about your experiences of support from your family and friends

c) Family and friends support
  - Can you tell me who, if anyone, offers you support with regards to your self‐harm? (eg family, friends, third sector, statutory services, Internet support groups)
  - How have they offered you support and how helpful has this been? In what ways?
  - Do you receive any sort of periodical support for your self‐harm? Could you please describe it? How helpful has this been? In what ways?

Are there other sorts of support that you would find helpful, or that you think other people might find helpful? Please say more….

*Closure*

Reflection and wrapping up

- Is there anything else you would like to add?
- How have you found today's interview? Any issues arising needing support?
- Are you happy to have a second interview?

Check consent

*END*
